# Testing for homogeneity of gametic disequilibrium across strata

**DOI:** 10.1186/1471-2156-8-85

**Published:** 2007-12-20

**Authors:** Xiaolin Yin, Wenqing Ma, Manlai Tang, Jianhua Guo

**Affiliations:** 1 Key Laboratory for Applied Statistics of MOE and School of Mathematics and Statistics, Northeast Normal University, Changchun 130024, China; 2 Department of Mathematics, Hong Kong Baptist University, Hong Kong, China

## Abstract

**Background:**

Assessing the non-random associations of alleles at different loci, or gametic disequilibrium, can provide clues about aspects of population histories and mating behavior and can be useful in locating disease genes. For gametic data which are available from several strata with different allele probabilities, it is necessary to verify that the strata are homogeneous in terms of gametic disequilibrium.

**Results:**

Using the likelihood score theory generalized to nuisance parameters we derive a score test for homogeneity of gametic disequilibrium across several independent populations. Simulation results demonstrate that the empirical type I error rates of our score homogeneity test perform satisfactorily in the sense that they are close to the pre-chosen 0.05 nominal level. The associated power and sample size formulae are derived. We illustrate our test with a data set from a study of the cystic fibrosis transmembrane conductance regulator gene.

**Conclusion:**

We propose a large-sample homogeneity test on gametic disequilibrium across several independent populations based on the likelihood score theory generalized to nuisance parameters. Our simulation results show that our test is more reliable than the traditional test based on the Fisher's test of homogeneity among correlation coefficients.

## Background

Measuring gametic disequilibrium can provide important information about aspects of population histories and mating behavior [1] and can be useful in locating disease genes [2]. The term gametic disequilibrium is used in this article instead of the traditional term linkage disequilibrium to measure the extent of non-random association because such non-random association may be present between unlinked loci [3]. Various measures of gametic disequilibrium have been proposed [4-6], ranging from pairs of diallelic loci model to multiple multiallelic loci model. In this article, we consider the gametic disequilibrium which is defined as the difference between the gametic probability and its expected probability under the assumption of no statistical association of alleles, and the gametic disequilibrium calculations are based on two-allele, two-locus model [7].

Consider two loci, *A *and *B*, each having two possible alleles (*A*_0_, *A*_1_) and (*B*_0_, *B*_1_), respectively. With two loci and two alleles, there are four possible gametes, namely, *A*_0_*B*_0_, *A*_0_*B*_1_, *A*_1_*B*_0 _and *A*_1_*B*_1_. The gametic disequilibrium between the two loci is defined by

$$D=p_{A_{1}B_{1}}-p_{A_{1}}p_{B_{1}},$$

where $p_{A_{i}}$ and $p_{B_{j}}$ denote the allele probabilities of *A*_*i *_and *B*_*j*_, $p_{A_{i}B_{j}}$ denotes the gamete probability of *A*_*i*_*B*_*j*_, *i*, *j *= 0, 1. Suppose that the gametic data are available from *K *strata and let *p*_*ijk *_denote the gametic probability of array of *A*_*i*_*B*_*j *_for the *k*-th stratum, *i*, *j *= 0, 1; *k *= 1,...,*K*, ∑_*i*,*j*_*p*_*ijk *_= 1 for each k. According to the relationship between allelic probability and gametic probability, the allele probabilities of *A*_0_, *A*_1_, *B*_0 _and *B*_1 _are derived as *p*_0+*k*_, *p*_1+*k*_, *p*_+0*k *_and *p*_+1*k*_, respectively. Here "+" denote the summation over 0 and 1, for example, *p*_0+*k *_= *p*_00*k *_+ *p*_01*k*_. For stratum k (*k *= 1,...,*K*), the gametic disequilibrium is calculated as

*D*_*k *_= *p*_11*k *_- *p*_1+*k*_*p*_+1*k*_.

It is easy to show that *D*_*k *_is bounded by

*D*_*k*,*min *_≤ *D*_*k *_≤ *D*_*k*,*max*_,

where *D*_*k*,*min *_= -min{*p*_1+*k*_*p*_+1*k*_, *p*_0+*k*_*p*_+0*k*_}, *D*_*k*,*max *_= min{*p*_1+*k*_*p*_+0*k*_, *p*_0+*k*_*p*_+1*k*_}. Testing for the homogeneity of gametic disequilibrium among strata can be informative in discriminating among the evolutionary agents generating them in natural population [8]. Detecting gametic disequilibrium can be informative in mapping gene and providing meaningful clues of population evolution. Combining the evidence of gametic disequilibrium across several strata may be more sufficient to support the clues, in contrast to analysis with each strata. In this case, it is crucial to test the homogeneity of gametic disequilibrium across strata before combining the data. For this purpose, it is interesting to consider the following hypothesis

*H*_0 _: *D*_1 _= ⋯ = *D*_*K*_   versus   *H*_1 _: *D*_*i *_≠ *D*_*j *_for at least a pair *i *≠ *j*.

Weir [9] recommended a homogeneity test on gametic disequilibrium, based on Fisher's test of homogeneity among correlation coefficients [10]. In his method, the gametic disequilibrium *D*_*k *_is first transformed to a correlation coefficient *r*_*k *_by *r*_*k *_= *D*_*k*_/$\sqrt{p_{1+k}p_{0+k}p_{+1k}p_{+0k}}$, *r*_*k *_is then transformed to a normal variable *z*_*k *_by Fisher's *z *transformation, and a weighted sum of squares of the *z *values which has *χ*^2 ^distribution with *K *- 1 degrees of freedom is finally proposed for testing homogeneity of gametic disequilibrium. As pointed out by Zapata and Alvarez [8], this test is actually for homogeneity of *r *values instead of *D *values. They may not be equivalent when the allele probabilities are different across strata. Instead, Zapata and Alvarez [8] suggested the use of the normalized difference *D' *[11]. Specifically, ${D^{\prime }}_{k}$ is the ratio of *D*_*k *_to *D*_*k*,*max *_when *D*_*k *_> 0, or the ratio of *D*_*k *_to -*D*_*k*,*min *_when *D*_*k *_< 0. Zapata and Alvarez obtained the bias-corrected confidence interval for each *D' *value across strata via the bootstrap method. Hence, acceptance or rejection of homogeneity of *D' *values can be determined by evaluating the obtained confidence intervals. For the example considered in Zapata and Alvarez [8], there is no intersection for the confidence intervals obtained from all strata. Hence, one has evidence to reject the null hypothesis of homogeneity. Unfortunately, Zapata and Alvarez [8] did not discuss the decision rules for cases such as intersections exist but the extent are different. Hence, no rigorous rule based on this confidence interval approach was proposed and this makes their method less practicable. However, no rigorous rule based on this confidence interval approach was proposed and this makes their method less practicable. It should be noted that the homogeneity test of either *r *values or *D' *values is not equivalent to the homogeneity test of *D *values. In particular, transformation *D' *only guarantees that the range of *D' *is [-1, 1]. However, there remains difficulties in interpreting the value of *D'*. Lewontin [11] noted that values of *D' *at different loci and in different populations tend to vary with the values of the allele probabilities, so that the problem of cross-locus and cross-population comparisons is not fully overcome by the use of *D'*. In this article, without doing any transformation, we develop an asymptotic homogeneity test directly based on *D *values via score method.

## Methods

### Homogeneity test

Let *x*_*ijk *_(*i*, *j *= 0, 1 and *k *= 1,⋯,*K*) be the number of the gamete *A*_*i*_*B*_*j *_in the *k*-th stratum with the total gametes being *n*_*k *_= *x*_00*k *_+ *x*_01*k *_+ *x*_10*k *_+ *s*_11*k*_. Let *M*(*n*_*k*_, {*p*_*ijk*_}) denote the quadrinomial distribution with parameter vector (*p*_00*k*_, *p*_01*k*_, *p*_10*k*_, *p*_11*k*_)*'*. Thus, we have {*x*_*ijk *_: *i*, *j *= 0, 1} ~ *M*(*n*_*k*_, {*p*_*ijk*_}) for *k *= 1,...,*K*. The homogeneity hypothesis in (1) is of interest in this article. Here, we assume that K is fixed and *n*_*k *_is sufficiently large for *k *= 1, 2,...,*K*. Noticing that *p*_00*k *_= *p*_0+*k*_*p*_+0*k *_+ *D*_*k*_, *p*_01*k *_= *p*_0+*k*_*p*_+1*k *_- *D*_*k*_, *p*_10*k *_= *p*_1+*k*_*p*_+0*k *_- *D*_*k*_, *p*_11*k *_= *p*_1+*k*_*p*_+1*k *_+ *D*_*k*_, the log-likelihood for the *k*-th stratum can be expressed in terms of *D*_*k*_, *p*_1+*k *_and *p*_+1*k *_(*k *= 1,....,*K*). That is,

$$\begin{array}{lll} l_{k}(D_{k},p_{1+k},p_{+1k}) & = & x_{00k}\ln(p_{0+k}p_{+0k}+D_{k})+x_{01k}ln(p_{0+k}p_{+1k}-D_{k})+\\ & & x_{10k}\ln(p_{1+k}p_{+0k}-D_{k})+x_{11k}ln(p_{1+k}p_{+1k}+D_{k}), \end{array}$$

where *p*_0+*k *_= 1 - *p*_1+*k*_, *p*_+0*k *_= 1 - *p*_+1*k*_. Let *D *denote the common gametic disequilibrium under *H*_0_, **p**_1+ _= (*p*_1+1_,...,*p*_1+*K*_)*' *and **p**_+1 _= (*p*_+11_,...,*p*_+1*K*_)*' *denote the nuisance parameter vectors. Under *H*_0_, the total log-likelihood for all *K *strata is given by

$$l(D,p_{1+},p_{+1})=\sum_{k=1}^{K}l_{k}(D,p_{1+k},p_{+1k}).$$

Hence, the efficient scores for the *k*-th stratum (i.e., the first order derivatives of *l*_*k*_(*D*, *p*_1+*k*_, *p*_+1*k*_) with respect to *D*, *p*_1+*k *_and *p*_+1*k*_) are given by

$$\begin{array}{lll} S_{kD}(D,p_{1+k},p_{+1k}) & = & \frac{\partial l_{k}}{\partial D}\\ & = & \frac{x_{00k}}{p_{0+k}p_{+0k}+D}-\frac{x_{01k}}{p_{0+k}p_{+1k}-D}-\frac{x_{10k}}{p_{1+k}p_{+0k}-D}+\frac{x_{11k}}{p_{1+k}p_{+1k}+D},\\ S_{kp_{1+k}}(D,p_{1+k},p_{+1k}) & = & \frac{\partial l_{k}}{\partial p_{1+k}}\\ & = & -\frac{x_{00k}p_{+0k}}{p_{0+k}p_{+0k}+D}-\frac{x_{01k}p_{+1k}}{p_{0+k}p_{+1k}-D}+\frac{x_{10k}p_{+0k}}{p_{1+k}p_{+0k}-D}+\frac{x_{11k}p_{+1k}}{p_{1+k}p_{+1k}+D},\\ S_{kp_{+1k}}(D,p_{1+k},p_{+1k}) & = & \frac{\partial l_{k}}{\partial p_{+1k}}\\ & = & -\frac{x_{00k}p_{0+k}}{p_{0+k}p_{+0k}+D}-\frac{x_{10k}p_{1+k}}{p_{1+k}p_{+0k}-D}+\frac{x_{01k}p_{0+k}}{p_{0+k}p_{+1k}-D}+\frac{x_{11k}p_{1+k}}{p_{1+k}p_{+1k}+D}. \end{array}$$

If $\hat{D}$, ${\hat{p}}_{1+}$ and ${\hat{p}}_{+1}$ are the maximum likelihood estimates (MLEs) of *D*, **p**_1+ _and **p**_+1 _under *H*_0_, respectively, then they satisfy the following 2*K *+ 1 equations:

$$\{\begin{array}{l} \sum_{k=1}^{K}S_{kD}(\hat{D},{\hat{p}}_{1+k},{\hat{p}}_{+1k})=0,\\ \begin{array}{cc} S_{kp_{1+k}}(\hat{D},{\hat{p}}_{1+k},{\hat{p}}_{+1k})=0, & k=1,2,\cdots ,K, \end{array}\\ \begin{array}{cc} S_{kp_{+1k}}(\hat{D},{\hat{p}}_{1+k},{\hat{p}}_{+1k})=0, & k=1,2,\cdots ,K. \end{array} \end{array}$$

Variances and covariances for the efficient scores are given by

$$\begin{array}{lll} I_{kDD} & = & Var(S_{kD}(D,p_{1+k},p_{+1k}))\\ & = & n_{k}[\frac{p_{0+k}}{\left(p_{0+k}p_{+0k}+D)(p_{0+k}p_{+1k}-D\right)}+\frac{p_{1+k}}{\left(p_{1+k}p_{+0k}-D)(p_{1+k}p_{+1k}+D\right)}],\\ I_{kp_{1+k}p_{1+k}} & = & Var(S_{kp_{1+k}}(D,p_{1+k},p_{+1k}))\\ & = & n_{k}[\frac{p_{+0k}^{3}}{\left(p_{0+k}p_{+0k}+D)(p_{1+k}p_{+0k}-D\right)}+\frac{p_{+1k}^{3}}{\left(p_{0+k}p_{+1k}-D)(p_{1+k}p_{+1k}+D\right)}],\\ I_{kp_{+1k}p_{+1k}} & = & Var(S_{kp_{+1k}}(D,p_{1+k},p_{+1k}))\\ & = & n_{k}[\frac{p_{0+k}^{3}}{\left(p_{0+k}p_{+0k}+D)(p_{0+k}p_{+1k}-D\right)}+\frac{p_{1+k}^{3}}{\left(p_{1+k}p_{+0k}-D)(p_{1+k}p_{+1k}+D\right)}],\\ I_{kDp_{1+k}} & = & Cov(S_{kD}(D,p_{1+k},p_{+1k}),S_{kp_{1+k}}(D,p_{1+k},p_{+1k}))\\ & = & n_{k}[\frac{p_{+1k}^{2}}{\left(p_{0+k}p_{+1k}-D)(p_{1+k}p_{+1k}+D\right)}-\frac{p_{+0k}^{2}}{\left(p_{0+k}p_{+0k}+D)(p_{1+k}p_{+0k}-D\right)}],\\ I_{kDp_{+1k}} & = & Cov(S_{kD}(D,p_{1+k},p_{+1k}),S_{kp_{+1k}}(D,p_{1+k},p_{+1k}))\\ & = & n_{k}[\frac{p_{1+k}^{2}}{\left(p_{1+k}p_{+0k}-D)(p_{1+k}p_{+1k}+D\right)}-\frac{p_{0+k}^{2}}{\left(p_{0+k}p_{+0k}+D)(p_{0+k}p_{+1k}-D\right)}],\\ I_{kp_{1+k}p_{+1k}} & = & Cov(S_{kp_{1+k}}(D,p_{1+k},p_{+1k}),S_{kp_{+1k}}(D,p_{1+k},p_{+1k}))\\ & = & -n_{k}D[\frac{p_{0+k}}{\left(p_{0+k}p_{+0k}+D)(p_{0+k}p_{+1k}-D\right)}+\frac{p_{1+k}}{\left(p_{1+k}p_{+0k}-D)(p_{1+k}p_{+1k}+D\right)}]. \end{array}$$

Denote

$$I_{kD|p_{1+k}p_{+1k}}=I_{kDD}-(I_{kDp_{1+k}},I_{kDp_{+1k}}){\left(\begin{array}{cc} I_{kp_{1+k}p_{1+k}} & I_{kp_{1+k}p_{+1k}}\\ I_{kp_{1+k}p_{+1k}} & I_{kp_{+1k}p_{+1k}} \end{array}\right)}^{-1}(I_{kDp_{1+k}},I_{kDp_{+1k}}{)}^{\prime }.$$

Hence, the likelihood score test for the homogeneity hypothesis *H*_0 _: *D*_1 _= ⋯ = *D*_*K *_is given by

$$X^{2}=\sum_{k=1}^{K}\frac{S_{kD}^{2}(\hat{D},{\hat{p}}_{1+k},{\hat{p}}_{+1k})}{I_{kD|p_{1+k}p_{+1k}}(\hat{D},{\hat{p}}_{1+k},{\hat{p}}_{+1k})},$$

which asymptotically follows the chi-square distribution with *K *- 1 degrees of freedom under *H*_0_.

Unfortunately, $\hat{D}$, ${\hat{p}}_{1+}$ and ${\hat{p}}_{+1}$ cannot be expressed in a closed form and this makes the likelihood score test *X*^2 ^less appealing in practice. To overcome this issue, applying the theory of homogeneity score test extended to nuisance parameters [12] we propose the following modified score statistic

$$X^{2*}=\sum_{k=1}^{K}\frac{S_{kD}^{2}(D^{*},p_{1+k}^{*},p_{+1k}^{*})}{I_{kD|p_{1+k}p_{+1k}}(D^{*},p_{1+k}^{*},p_{+1k}^{*})}-\frac{{\left[\sum_{k=1}^{K}S_{kD}(D^{*},p_{1+k}^{*},p_{+1k}^{*})\right]}^{2}}{\sum_{k=1}^{K}I_{kD|p_{1+k}p_{+1k}}(D^{*},p_{1+k}^{*},p_{+1k}^{*})},$$

where D*, $p_{1+}^{*}$ and $p_{+1}^{*}$ are any consistent estimators of *D*, **p**_1+ _and **p**_+1_, respectively. To this end, we choose D* to be $\sum_{k=1}^{K}(\frac{x_{00k}x_{11k}}{x_{01k}x_{10k}}-1)/\sum_{k=1}^{K}\frac{n_{k}^{2}}{x_{01k}x_{10k}}$, and $p_{1+k}^{*}$ and $p_{+1k}^{*}$ be the solutions to the following equations

$$\{\begin{array}{c} S_{kp_{1+k}}(D^{*},p_{1+k},p_{+1k})\equiv -\frac{x_{00k}p_{+0k}}{p_{0+k}p_{+0k}+D^{*}}-\frac{x_{01k}p_{+1k}}{p_{0+k}p_{+1k}-D^{*}}+\frac{x_{10k}p_{+0k}}{p_{1+k}p_{+0k}-D^{*}}+\frac{x_{11k}p_{+1k}}{p_{1+k}p_{+1k}+D^{*}}=0,\\ S_{kp_{+1k}}(D^{*},p_{1+k},p_{+1k})\equiv -\frac{x_{00k}p_{0+k}}{p_{0+k}p_{+0k}+D^{*}}-\frac{x_{10k}p_{1+k}}{p_{1+k}p_{+0k}-D^{*}}+\frac{x_{01k}p_{0+k}}{p_{0+k}p_{+1k}-D^{*}}+\frac{x_{11k}p_{1+k}}{p_{1+k}p_{+1k}+D^{*}}=0, \end{array}$$

or equivalently the following quartic polynomial equations,

$$\{\begin{array}{l} a_{0}+a_{1}p_{+1k}+a_{2}p_{+1k}^{2}+a_{3}p_{+1k}^{3}+a_{4}p_{+1k}^{4}=0,\\ b_{0}+b_{1}p_{1+k}+b_{2}p_{1+k}^{2}+b_{3}p_{1+k}^{3}+b_{4}p_{1+k}^{4}=0, \end{array}$$

where

$$\begin{array}{lll} a_{0} & = & [x_{+0k}(p_{1+k}-D^{*})-x_{10k}]{\left(D^{*}\right)}^{2},\\ a_{1} & = & (n_{k}+x_{+0k})D^{*}p_{1+k}^{2}-[2(n_{k}+x_{+0k})D^{*}+n_{k}+2x_{10k}]D^{*}p_{1+k}+\\ & & [n_{k}{\left(D^{*}\right)}^{2}+(n_{k}+2x_{10k})D^{*}+x_{10k}]D^{*},\\ a_{2} & = & n_{k}p_{1+k}^{3}-[(4n_{k}+x_{+0k})D^{*}+n_{k}+x_{1+k}]p_{1+k}^{2}+[3n_{k}{\left(D^{*}\right)}^{2}+\\ & & (3n_{k}+4x_{10k}+2x_{11k})D^{*}+x_{1+k}]p_{1+k}-[(n_{k}+x_{1+k})D^{*}+2x_{10k}+x_{11k}]D^{*},\\ a_{3} & = & -2n_{k}p_{1+k}^{3}+[3n_{k}D^{*}+2(n_{k}+x_{1+k})]p_{1+k}^{2}-2[(n_{k}+x_{1+k})D^{*}+x_{1+k}]p_{1+k}+x_{1+k}D^{*},\\ a_{4} & = & n_{k}p_{1+k}^{3}-(n_{k}+x_{1+k})p_{1+k}^{2}+x_{1+k}p_{1+k},\\ b_{0} & = & [x_{0+k}(p_{+1k}-D^{*})-x_{01k}]{\left(D^{*}\right)}^{2},\\ b_{1} & = & (n_{k}+x_{0+k})D^{*}p_{+1k}^{2}-[2(n_{k}+x_{0+k})D^{*}+n_{k}+2x_{01k}]D^{*}p_{+1k}+\\ & & [n_{k}{\left(D^{*}\right)}^{2}+(n_{k}+2x_{01k})D^{*}+x_{01k}]D^{*},\\ b_{2} & = & n_{k}p_{+1k}^{3}-[(4n_{k}+x_{+1k})D^{*}+n_{k}+x_{+1k}]p_{+1k}^{2}+[3n_{k}{\left(D^{*}\right)}^{2}+\\ & & (3n_{k}+4x_{01k}+2x_{11k})D^{*}+x_{+1k}]p_{+1k}-[(n_{k}+x_{+1k})D^{*}+2x_{01k}+x_{11k}]D^{*},\\ b_{3} & = & -2n_{k}p_{+1k}^{3}+[3n_{k}D^{*}+2(n_{k}+x_{+1k})]p_{+1k}^{2}-2[(n_{k}+x_{+1k})D^{*}+x_{+1k}]p_{+1k}+x_{+1k}D^{*},\\ b_{4} & = & n_{k}p_{+1k}^{3}-(n_{k}+x_{+1k})p_{+1k}^{2}+x_{+1k}p_{+1k}. \end{array}$$

Here, D* is analogous to the well-known Mantel-Haenszel estimator [13]. It is a consistent estimator to *D*. In general, it is not an efficient estimator to *D*. The proof of consistency and the conditions for achieving asymptotic efficiency for D* is presented in Appendix. We notice that the calculation of $I_{kD|p_{1+k}p_{+1k}}$ in (2) is quite tedious. Nonetheless, it is easy to show that $I_{kD|p_{1+k}p_{+1k}}$ is simply given by *n*_*k*_/*w*_*k*_(*D*, *p*_1+*k*_, *p*_+1*k*_) with $w_{k}(D,p_{1+k},p_{+1k})=p_{11k}p_{00k}^{2}+p_{10k}p_{01k}^{2}+p_{01k}p_{10k}^{2}+p_{00k}p_{11k}^{2}-4D^{2}$ (see Appendix for the proof). It can be shown that *X*^2* ^has an asymptotic chi-square distribution with *K *- 1 degrees of freedom under *H*_0_. Therefore, the homogeneity hypothesis *H*_0 _is rejected at level *α *when *X*^2* ^≥ $\chi_{K-1,(1-\alpha )}^{2}$, where $\chi_{K-1,(1-\alpha )}^{2}$ is the 100 × (1 - *α*) percentile point of the chi-square distribution with *K *- 1 degrees of freedom. Finally, it is noteworthy that if the consistent estimators of *D*, **p**_1+ _and **p**_+1 _are the constrained MLEs under *H*_0 _then the second term of (2) vanishes, since $\sum_{k=1}^{K}S_{kD}(D^{*},p_{1+k}^{*},p_{+1k}^{*})=0$, and (2) reduces to the likelihood score statistic.

### Asymptotic power and sample size

We will present the asymptotic power and sample size formulae based on *X*^2* ^[14]. For this purpose, we assume *n*_*k *_= *na*_*k *_for some *n *and *a*_*k *_> 0. Let ${\bar{D}}_{k}$, ${\bar{p}}_{1+k}$ and ${\bar{p}}_{+1k}$ be the true parameter values for *D*_*k*_, *p*_1+*k *_and *p*_+1*k *_under *H*_1_, where *k *= 1, 2,⋯,*K *and ${\bar{D}}_{k}\neq {\bar{D}}_{j}$ for at least a pair *k *≠ *j*. Thus, the asymptotic power for the homogeneity score test *X*^2* ^at *α *level is given by

$$Pr(X^{2*}\geq \chi_{K-1,(1-\alpha )}^{2}|H_{1})=Pr(\chi_{K-1}^{2}(\Delta )\geq \chi_{K-1,(1-\alpha )}^{2},$$

where $\chi_{K-1}^{2}(\Delta )$ denotes the non-central chi-square distribution with *K *- 1 degrees of freedom with the non-centrality parameter being

$$\begin{array}{lll} \Delta & = & n\{\sum_{k=1}^{K}\frac{a_{k}{\left(\frac{{\bar{p}}_{0+k}{\bar{p}}_{+0k}+{\bar{D}}_{k}}{p_{0+k}p_{+0k}+d}-\frac{{\bar{p}}_{0+k}{\bar{p}}_{+1k}-{\bar{D}}_{k}}{p_{0+k}p_{+1k}-d}-\frac{{\bar{p}}_{1+k}{\bar{p}}_{+0k}-{\bar{D}}_{k}}{p_{1+k}p_{+0k}-d}+\frac{{\bar{p}}_{1+k}{\bar{p}}_{+1k}+{\bar{D}}_{k}}{p_{1+k}p_{+1k}+d}\right)}^{2}}{1/w_{k}(d,p_{1+k},p_{+1k})}-\\ & & \frac{\{\sum_{k=1}^{K}a_{k}{\left(\frac{{\bar{p}}_{0+k}{\bar{p}}_{+0k}+{\bar{D}}_{k}}{p_{0+k}p_{+0k}+d}-\frac{{\bar{p}}_{0+k}{\bar{p}}_{+1k}-{\bar{D}}_{k}}{p_{0+k}p_{+1k}-d}-\frac{{\bar{p}}_{1+k}{\bar{p}}_{+0k}-{\bar{D}}_{k}}{p_{1+k}p_{+0k}-d}+\frac{{\bar{p}}_{1+k}{\bar{p}}_{+1k}+{\bar{D}}_{k}}{p_{1+k}p_{+1k}+d}\right)}^{2}}{\sum_{k=1}^{K}\left[a_{k}/w_{k}(d,p_{1+k},p_{+1k})\right]}\}, \end{array}$$

where $d=\sum_{k=1}^{K}[\frac{\left({\bar{p}}_{0+k}{\bar{p}}_{+0k}+{\bar{D}}_{k})({\bar{p}}_{1+k}{\bar{p}}_{+1k}+{\bar{D}}_{k}\right)}{\left({\bar{p}}_{0+k}{\bar{p}}_{+1k}-{\bar{D}}_{k})({\bar{p}}_{1+k}{\bar{p}}_{+0k}-{\bar{D}}_{k}\right)}-1]/\sum_{k=1}^{K}\frac{1}{\left({\bar{p}}_{0+k}{\bar{p}}_{+1k}+{\bar{D}}_{k})({\bar{p}}_{1+k}{\bar{p}}_{+0k}-{\bar{D}}_{k}\right)}$, ${\bar{p}}_{0+k}=1-{\bar{p}}_{1+k}$, ${\bar{p}}_{+0k}=1-{\bar{p}}_{+1k}$, *p*_1+*k *_and *p*_+1*k *_are the solutions of the following equations

$$\{\begin{array}{c} {\bar{a}}_{0}+{\bar{a}}_{1}p_{+1k}+{\bar{a}}_{2}p_{+1k}^{2}+{\bar{a}}_{3}p_{+1k}^{3}+{\bar{a}}_{4}p_{+1k}^{4}=0,\\ {\bar{b}}_{0}+{\bar{b}}_{1}p_{1+k}+{\bar{b}}_{2}p_{1+k}^{2}+{\bar{b}}_{3}p_{1+k}^{3}+{\bar{b}}_{4}p_{1+k}^{4}=0, \end{array}$$

where

$$\begin{array}{lll} {\bar{a}}_{0} & = & [{\bar{p}}_{+0k}(p_{1+k}-d)-{\bar{p}}_{10k}]d^{2},\\ {\bar{a}}_{1} & = & (1+{\bar{p}}_{+0k})dp_{1+k}^{2}-[2(1+{\bar{p}}_{+0k})d+1+2{\bar{p}}_{10k}]dp_{1+k}+\\ & & [d^{2}+(1+2{\bar{p}}_{10k})d+{\bar{p}}_{10k}]d,\\ {\bar{a}}_{2} & = & p_{1+k}^{3}-[(4+{\bar{p}}_{+0k})d+1+{\bar{p}}_{1+k}]p_{1+k}^{2}+[3d^{2}+\\ & & (3+4{\bar{p}}_{10k}+2{\bar{p}}_{11k})d+{\bar{p}}_{1+k}]p_{1+k}-[(1+{\bar{p}}_{1+k})d+2{\bar{p}}_{10k}+{\bar{p}}_{11k}]d,\\ {\bar{a}}_{3} & = & -2p_{1+k}^{3}+[3d+2(1+{\bar{p}}_{1+k})]p_{1+k}^{2}-2[(1+{\bar{p}}_{1+k})d+{\bar{p}}_{1+k}]p_{1+k}+{\bar{p}}_{1+k}d,\\ {\bar{a}}_{4} & = & p_{1+k}^{3}-(1+{\bar{p}}_{1+k})p_{1+k}^{2}+{\bar{p}}_{1+k}p_{1+k},\\ {\bar{b}}_{0} & = & [{\bar{p}}_{0+k}(p_{+1k}-d)-{\bar{p}}_{01k}]d^{2},\\ {\bar{b}}_{1} & = & (1+{\bar{p}}_{0+k})dp_{+1k}^{2}-[2(1+{\bar{p}}_{0+k})d+n_{k}+2{\bar{p}}_{01k}]dp_{+1k}+\\ & & [d^{2}+(1+2{\bar{p}}_{01k})d+{\bar{p}}_{01k}]d,\\ {\bar{b}}_{2} & = & p_{+1k}^{3}-[(4+{\bar{p}}_{+1k})d+1+{\bar{p}}_{+1k}]p_{+1k}^{2}+[3d^{2}+\\ & & (3+4{\bar{p}}_{01k}+2{\bar{p}}_{11k})d+{\bar{p}}_{+1k}]p_{+1k}-[(1+{\bar{p}}_{+1k})d+2{\bar{p}}_{01k}+{\bar{p}}_{11k}]d,\\ {\bar{b}}_{3} & = & -2p_{+1k}^{3}+[3d+2(1+{\bar{p}}_{+1k})]p_{+1k}^{2}-2[(1+{\bar{p}}_{+1k})d+{\bar{p}}_{+1k}]p_{+1k}+{\bar{p}}_{+1k}d,\\ {\bar{b}}_{4} & = & p_{+1k}^{3}-(1+{\bar{p}}_{+1k})p_{+1k}^{2}+{\bar{p}}_{+1k}p_{+1k}. \end{array}$$

The desirable sample size *n *required to attain the power at 1 - *β *with ${\bar{D}}_{k}$, ${\bar{p}}_{1+k}$ and ${\bar{p}}_{+1k}$ being the true parameter values for *D*_*k*_, *p*_1+*k *_and *p*_+1*k *_under the alternative *H*_1 _at nominal level *α *can be found by the relation

$$\chi_{K-1,\beta }^{2}(\Delta )=\chi_{K-1,(1-\alpha )}^{2},$$

where $\chi_{K-1,\beta }^{2}(\Delta )$ is the 100 × *β *percentile point of the non-central chi-square distribution with *K *- 1 degrees of freedom and non-centrality parameter Δ. The sample size *n *can be readily obtained by solving the above equation.

### Availability and requirements

We have implemented the test procedures for computing our score statistic *X*^2* ^in a Matlab project. Project name: gametic disequilibrium homogeneity score test (GDHST); Project home page: ; Operating system: Windows XP; Programming language: Matlab 6.1; Licence: GNU GPL.

## Results

### Simulation results

To evaluate the performance of our proposed homogeneity score test, we include the homogeneity test recommended by Weir [9] in our comparison study. The corresponding test statistic for homogeneity is given by

$$T^{2}=\sum_{k=1}^{K}(n_{k}-3){\left(z_{k}-\bar{z}\right)}^{2},$$

where *K *is the total number of strata, *n*_*k *_is the total gamete number in stratum *k*, $z_{k}=\frac{1}{2}\ln(\frac{1+r_{k}}{1-r_{k}})$ is the Fisher's z transformation with $r_{k}=\frac{n_{k}x_{11k}-x_{1+k}x_{+1k}}{\sqrt{x_{0+k}x_{+0k}x_{1+k}x_{+1k}}}$ and (*x*_00*k*_, *x*_01*k*_, *x*_10*k*_, *x*_11*k*_)*' *being the number of the gamete array in the *k*-th stratum, and $\bar{z}$ is the average of the *z*_*k *_values.

We investigate the performance of *X*^2* ^and *T*^2 ^in terms of type I error rate and power. For type I error rates, we consider both equal and unequal allele probabilities varying from 0.1 to 0.5 across (K = 3 and 5) strata with equal sample sizes (*n*_*k *_= 50, 100 and 200) for *k *= 1,...,*K *and common disequilibrium (*D *= $\frac{1}{2}$*D*_*min*_, 0 and $\frac{1}{2}$*D*_*max*_), where *D*_*min *_= *max*{*D*_1,*min*_,...,*D*_*K*,*min*_}, *D*_*max *_= *min*{*D*_1,*max*_,...,*D*_*K*,*max*_}.

Monte Carlo simulations with 5,000 repetitions at 0.05 nominal level are summarized in Table 1, 2, 3, 4. Table 1 shows the performance of empirical type I error rates for *X*^2* ^and *T*^2 ^with equal allele probabilities across K = 3 strata. We observe the following.

**Table 1:** Empirical type I error rates for *X*^2* ^and *T*^2 ^for equal allele probabilities across *K *= 3 strata under *H*_0_

n	D	**p**_1+_	**p**_+1_	*X*^2*^	*T*^2^
50, 50, 50	-0.125	0.5, 0.5, 0.5	0.5, 0.5, 0.5	0.047	0.110
	0.0			0.055	0.052
	0.125			0.076	0.116
	-0.075	0.5, 0.5, 0.5	0.3, 0.3, 0.3	0.051	0.061
	0.0			0.053	0.049
	0.075			0.075	0.063
	-0.045	0.3, 0.3, 0.3	0.3, 0.3, 0.3	0.042	0.021
	0.0			0.044	0.053
	0.105			0.100	0.167
	-0.025	0.5, 0.5, 0.5	0.1, 0.1, 0.1	0.061	0.036
	0.0			0.067	0.041
	0.025			0.089	0.029
	-0.015	0.3, 0.3, 0.3	0.1, 0.1, 0.1	0.024	0.017
	0.0			0.025	0.047
	0.035			0.104	0.117
	-0.005	0.1, 0.1, 0.1	0.1, 0.1, 0.1	0.031	0.024
	0.0			0.024	0.087
	0.045			0.413	0.515
100, 100, 100	-0.125	0.5, 0.5, 0.5	0.5, 0.5, 0.5	0.049	0.106
	0.0			0.052	0.051
	0.125			0.065	0.112
	-0.075	0.5, 0.5, 0.5	0.3, 0.3, 0.3	0.051	0.059
	0.0			0.049	0.051
	0.075			0.055	0.063
	-0.045	0.3, 0.3, 0.3	0.3, 0.3, 0.3	0.046	0.024
	0.0			0.048	0.051
	0.105			0.048	0.156
	-0.025	0.5, 0.5, 0.5	0.1, 0.1, 0.1	0.053	0.029
	0.0			0.048	0.047
	0.025			0.089	0.030
	-0.015	0.3, 0.3, 0.3	0.1, 0.1, 0.1	0.026	0.013
	0.0			0.029	0.048
	0.035			0.075	0.109
	-0.005	0.1, 0.1, 0.1	0.1, 0.1, 0.1	0.012	0.014
	0.0			0.013	0.057
	0.045			0.278	0.474
200, 200, 200	-0.125	0.5, 0.5, 0.5	0.5, 0.5, 0.5	0.050	0.050
	0.0			0.052	0.048
	0.125			0.058	0.112
	-0.075	0.5, 0.5, 0.5	0.3, 0.3, 0.3	0.050	0.057
	0.0			0.050	0.049
	0.075			0.054	0.058
	-0.045	0.3, 0.3, 0.3	0.3, 0.3, 0.3	0.049	0.025
	0.0			0.049	0.049
	0.105			0.052	0.156
	-0.025	0.5, 0.5, 0.5	0.1, 0.1, 0.1	0.053	0.030
	0.0			0.050	0.051
	0.025			0.052	0.032
	-0.015	0.3, 0.3, 0.3	0.1, 0.1, 0.1	0.044	0.014
	0.0			0.044	0.051
	0.035			0.045	0.105
	-0.005	0.1, 0.1, 0.1	0.1, 0.1, 0.1	0.020	0.010
	0.0			0.020	0.049
	0.045			0.098	0.463

The empirical type I error rate which is significant for the two-sided t-test at the nominal level of 0.05 is marked with underline.

**Table 2:** Empirical type I error rates for *X*^2* ^and *T*^2 ^for unequal allele probabilities across *K *= 3 strata under *H*_0_

n	D	**p**_1+_	**p**_+1_	*X*^2*^	*T*^2^
50, 50, 50	-0.045	0.5, 0.4, 0.3	0.5, 0.4, 0.3	0.048	0.044
	0.0			0.049	0.051
	0.105			0.071	0.149
	-0.015	0.5, 0.4, 0.3	0.5, 0.3, 0.1	0.046	0.037
	0.0			0.046	0.051
	0.035			0.066	0.105
	-0.005	0.5, 0.3, 0.1	0.5, 0.3, 0.1	0.063	0.036
	0.0			0.053	0.052
	0.045			0.100	0.474
	-0.015	0.5, 0.4, 0.3	0.3, 0.2, 0.1	0.040	0.032
	0.0			0.042	0.049
	0.035			0.074	0.101
	-0.005	0.5, 0.3, 0.1	0.3, 0.2, 0.1	0.056	0.033
	0.0			0.048	0.054
	0.045			0.108	0.452
	-0.005	0.3, 0.2, 0.1	0.3, 0.2, 0.1	0.048	0.031
	0.0			0.041	0.053
	0.045			0.123	0.452
100, 100, 100	-0.045	0.5, 0.4, 0.3	0.5, 0.4, 0.3	0.051	0.048
	0.0			0.050	0.050
	0.105			0.055	0.162
	-0.015	0.5, 0.4, 0.3	0.5, 0.3, 0.1	0.047	0.043
	0.0			0.046	0.050
	0.035			0.054	0.150
	-0.005	0.5, 0.3, 0.1	0.5, 0.3, 0.1	0.064	0.037
	0.0			0.052	0.051
	0.045			0.059	0.658
	-0.015	0.5, 0.4, 0.3	0.3, 0.2, 0.1	0.044	0.035
	0.0			0.047	0.051
	0.035			0.051	0.124
	-0.005	0.5, 0.3, 0.1	0.3, 0.2, 0.1	0.055	0.033
	0.0			0.052	0.053
	0.045			0.063	0.623
	-0.005	0.3, 0.2, 0.1	0.3, 0.2, 0.1	0.055	0.033
	0.0			0.043	0.051
	0.045			0.061	0.593
200, 200, 200	-0.045	0.5, 0.4, 0.3	0.5, 0.4, 0.3	0.050	0.052
	0.0			0.050	0.052
	0.105			0.053	0.211
	-0.015	0.5, 0.4, 0.3	0.5, 0.3, 0.1	0.049	0.049
	0.0			0.048	0.049
	0.035			0.049	0.220
	-0.005	0.5, 0.3, 0.1	0.5, 0.3, 0.1	0.058	0.037
	0.0			0.051	0.050
	0.045			0.048	0.860
	-0.015	0.5, 0.4, 0.3	0.3, 0.2, 0.1	0.048	0.043
	0.0			0.049	0.048
	0.035			0.050	0.190
	-0.005	0.5, 0.3, 0.1	0.3, 0.2, 0.1	0.056	0.035
	0.0			0.051	0.051
	0.045			0.053	0.829
	-0.005	0.3, 0.2, 0.1	0.3, 0.2, 0.1	0.051	0.034
	0.0			0.050	0.050
	0.045			0.049	0.791

The empirical type I error rate which is significant for the two-sided t-test at the nominal level of 0.05 is marked with underline.

**Table 3:** Empirical type I error rates for *X*^2* ^and *T*^2 ^for equal allele probabilities across *K *= 5 strata under *H*_0_

n	D	**p**_1+_	**p**_+1_	*X*^2*^	*T*^2^
50, 50, 50, 50, 50	-0.125	0.5, 0.5, 0.5, 0.5, 0.5	0.5, 0.5, 0.5, 0.5, 0.5	0.041	0.146
	0.0			0.053	0.048
	0.125			0.090	0.148
	-0.075	0.5, 0.5, 0.5, 0.5, 0.5	0.3, 0.3, 0.3, 0.3, 0.3	0.049	0.059
	0.0			0.053	0.051
	0.075			0.084	0.063
	-0.045	0.3, 0.3, 0.3, 0.3, 0.3	0.3, 0.3, 0.3, 0.3, 0.3	0.036	0.018
	0.0			0.039	0.048
	0.105			0.172	0.228
	-0.025	0.5, 0.5, 0.5, 0.5, 0.5	0.1, 0.1, 0.1, 0.1, 0.1	0.075	0.029
	0.0			0.071	0.038
	0.025			0.059	0.026
	-0.015	0.3, 0.3, 0.3, 0.3, 0.3	0.1, 0.1, 0.1, 0.1, 0.1	0.015	0.012
	0.0			0.028	0.043
	0.035			0.136	0.147
	-0.005	0.1, 0.1, 0.1, 0.1, 0.1	0.1, 0.1, 0.1, 0.1, 0.1	0.025	0.024
	0.0			0.026	0.079
	0.045			0.500	0.715
100, 100, 100, 100, 100	-0.125	0.5, 0.5, 0.5, 0.5, 0.5	0.5, 0.5, 0.5, 0.5, 0.5	0.046	0.135
	0.0			0.051	0.050
	0.125			0.068	0.141
	-0.075	0.5, 0.5, 0.5, 0.5, 0.5	0.3, 0.3, 0.3, 0.3, 0.3	0.050	0.059
	0.0			0.051	0.051
	0.075			0.054	0.059
	-0.045	0.3, 0.3, 0.3, 0.3, 0.3	0.3, 0.3, 0.3, 0.3, 0.3	0.046	0.020
	0.0			0.044	0.052
	0.105			0.051	0.212
	-0.025	0.5, 0.5, 0.5, 0.5, 0.5	0.1, 0.1, 0.1, 0.1, 0.1	0.063	0.030
	0.0			0.058	0.048
	0.025			0.057	0.029
	-0.015	0.3, 0.3, 0.3, 0.3, 0.3	0.1, 0.1, 0.1, 0.1, 0.1	0.026	0.009
	0.0			0.025	0.047
	0.035			0.088	0.137
	-0.005	0.1, 0.1, 0.1, 0.1, 0.1	0.1, 0.1, 0.1, 0.1, 0.1	0.016	0.007
	0.0			0.008	0.006
	0.045			0.476	0.667
200, 200, 200, 200, 200	-0.125	0.5, 0.5, 0.5, 0.5, 0.5	0.5, 0.5, 0.5, 0.5, 0.5	0.051	0.134
	0.0			0.049	0.049
	0.125			0.061	0.133
	-0.075	0.5, 0.5, 0.5, 0.5, 0.5	0.3, 0.3, 0.3, 0.3, 0.3	0.051	0.055
	0.0			0.049	0.050
	0.075			0.054	0.059
	-0.045	0.3, 0.3, 0.3, 0.3, 0.3	0.3, 0.3, 0.3, 0.3, 0.3	0.053	0.022
	0.0			0.050	0.051
	0.105			0.050	0.203
	-0.025	0.5, 0.5, 0.5, 0.5, 0.5	0.1, 0.1, 0.1, 0.1, 0.1	0.054	0.027
	0.0			0.048	0.049
	0.025			0.053	0.028
	-0.015	0.3, 0.3, 0.3, 0.3, 0.3	0.1, 0.1, 0.1, 0.1, 0.1	0.037	0.008
	0.0			0.037	0.049
	0.035			0.044	0.123
	-0.005	0.1, 0.1, 0.1, 0.1, 0.1	0.1, 0.1, 0.1, 0.1, 0.1	0.017	0.007
	0.0			0.018	0.053
	0.045			0.193	0.651

The empirical type I error rate which is significant for the two-sided t-test at the nominal level of 0.05 is marked with underline.

**Table 4:** Empirical type I error rates for *X*^2* ^and *T*^2 ^for unequal allele probabilities across *K *= 5 strata under *H*_0_

n	D	**p**_1+_	**p**_+1_	*X*^2*^	*T*^2^
50, 50, 50, 50, 50	-0.045	0.5, 0.4, 0.3, 0.4, 0.5	0.5, 0.4, 0.3, 0.4, 0.5	0.048	0.049
	0.0			0.048	0.049
	0.105			0.085	0.163
	-0.025	0.5, 0.4, 0.3, 0.4, 0.5	0.5, 0.4, 0.3, 0.2, 0.1	0.047	0.041
	0.0			0.057	0.054
	0.025			0.077	0.060
	-0.005	0.5, 0.4, 0.3, 0.2, 0.1	0.5, 0.4, 0.3, 0.2, 0.1	0.048	0.035
	0.0			0.042	0.057
	0.045			0.133	0.489
	-0.015	0.5, 0.4, 0.3, 0.4, 0.5	0.3, 0.2, 0.1, 0.2, 0.3	0.045	0.037
	0.0			0.045	0.053
	0.035			0.082	0.100
	-0.015	0.5, 0.4, 0.3, 0.2, 0.1	0.3, 0.2, 0.1, 0.2, 0.3	0.032	0.023
	0.0			0.034	0.048
	0.035			0.104	0.136
	-0.005	0.3, 0.2, 0.1, 0.2, 0.3	0.3, 0.2, 0.1, 0.2, 0.3	0.045	0.034
	0.0			0.035	0.057
	0.045			0.160	0.475
100, 100, 100, 100, 100	-0.045	0.5, 0.4, 0.3, 0.4, 0.5	0.5, 0.4, 0.3, 0.4, 0.5	0.050	0.051
	0.0			0.051	0.051
	0.105			0.060	0.190
	-0.025	0.5, 0.4, 0.3, 0.4, 0.5	0.5, 0.4, 0.3, 0.2, 0.1	0.050	0.050
	0.0			0.051	0.051
	0.025			0.062	0.067
	-0.005	0.5, 0.4, 0.3, 0.2, 0.1	0.5, 0.4, 0.3, 0.2, 0.1	0.050	0.037
	0.0			0.041	0.049
	0.045			0.058	0.653
	-0.015	0.5, 0.4, 0.3, 0.4, 0.5	0.3, 0.2, 0.1, 0.2, 0.3	0.046	0.040
	0.0			0.047	0.051
	0.035			0.054	0.118
	-0.015	0.5, 0.4, 0.3, 0.2, 0.1	0.3, 0.2, 0.1, 0.2, 0.3	0.037	0.027
	0.0			0.037	0.050
	0.035			0.060	0.168
	-0.005	0.3, 0.2, 0.1, 0.2, 0.3	0.3, 0.2, 0.1, 0.2, 0.3	0.047	0.035
	0.0			0.038	0.053
	0.045			0.060	0.610
200, 200, 200, 200, 200	-0.045	0.5, 0.4, 0.3, 0.4, 0.5	0.5, 0.4, 0.3, 0.4, 0.5	0.049	0.050
	0.0			0.048	0.049
	0.105			0.053	0.230
	-0.025	0.5, 0.4, 0.3, 0.4, 0.5	0.5, 0.4, 0.3, 0.2, 0.1	0.049	0.062
	0.0			0.051	0.050
	0.025			0.053	0.081
	-0.005	0.5, 0.4, 0.3, 0.2, 0.1	0.5, 0.4, 0.3, 0.2, 0.1	0.051	0.039
	0.0			0.043	0.048
	0.045			0.052	0.865
	-0.015	0.5, 0.4, 0.3, 0.4, 0.5	0.3, 0.2, 0.1, 0.2, 0.3	0.048	0.044
	0.0			0.049	0.049
	0.035			0.048	0.169
	-0.015	0.5, 0.4, 0.3, 0.2, 0.1	0.3, 0.2, 0.1, 0.2, 0.3	0.042	0.029
	0.0			0.045	0.053
	0.035			0.045	0.229
	-0.005	0.3, 0.2, 0.1, 0.2, 0.3	0.3, 0.2, 0.1, 0.2, 0.3	0.047	0.035
	0.0			0.040	0.053
	0.045			0.051	0.815

The empirical type I error rate which is significant for the two-sided t-test at the nominal level of 0.05 is marked with underline.

1. When *D *is large (i.e., $\frac{1}{2}$*D*_*max*_), both tests generally appear to be quite liberal (e.g., empirical size being 10 times of the nominal level), especially for small sample size (e.g., *n*_*k *_= 50) and small allele probability (e.g., **p**_1+ _= **p**_+1 _= (0.1, 0.1, 0.1)*'*). Such liberty in empirical size is more severe in *T*^2 ^than in our asymptotic homogeneity test *X*^2* ^and is significantly alleviated in *X*^2* ^when sample size increases. However, sample size increase does not alleviate the liberty of *T*^2 ^much. In fact, even for *n*_*k *_= 3200 for *k *= 1, 2, 3, *T*^2 ^is still very liberal for D = 0.045 with empirical type I errors rate being 0.456 (data are not shown).

2. For other settings, both tests perform quite satisfactorily in the sense that their empirical sizes are well controlled around the pre-chosen nominal level. In general, the larger the sample size, the closer the empirical type I error rate to the pre-chosen nominal level.

Table 2 reports the empirical size performance of *X*^2* ^and *T*^2 ^for unequal allele probabilities across K = 3 strata. We observe similar phenomena above. However, our asymptotic homogeneity test *X*^2* ^performs quite well in all settings under consideration for moderate to large sample sizes (i.e., *n*_*k *_= 100 and 200) while it is not the case for *T*^2^. For *T*^2^, the resultant empirical type I error rate can be extremely inflated even for large sample design (e.g., more than 17 times of the nominal level when *n*_*k *_= 200 (for *k *= 1, 2, 3), **p**_1+ _= **p**_+1 _= (0.5, 0.3, 0.1)*'*, and *D *= 0.045).

Table 3 and 4 shows the empirical type I error rate performance of *X*^2* ^and *T*^2 ^for *K *= 5. The parameter settings are similar to Table 1 and 2. According to the simulation results, liberty issue becomes more serious and larger sample sizes are required to attain similar performance when *K *increases from 3 to 5 under similar parameter settings.

Since many type I error rates for *X*^2* ^and *T*^2 ^are liberal in Tables 1 to 4. The two-sided t-test is conducted to determined if an empirical type I error rate is significantly different from the nominal lever of 0.05. The t-test statistics is

$$\sqrt{m-1}\frac{W-0.05}{\sqrt{W(1-W)}},$$

where m = 5000 and W represents the empirical type I error rate of *X*^2* ^or *T*^2^. Here, the t-test is almost identical to the z-test for the sample size is very large. Those empirical type I error rates which are significantly different from the nominal level of 0.05 are underlined in Tables 1 to 4. In Table 1, the total number of significant difference from the nominal level of 0.05 for *X*^2* ^and *T*^2 ^is 28 and 38, respectively. The pair (28, 38) can be further decomposed to (14, 14), (8, 13) and (6, 11) according to n = 50, 100 and 200. The decreasing rate of the number of empirical type I error rates which is significant different from the nominal level of 0.05 for *X*^2* ^is 14/18-6/18 = 44.4% as n increases from 50 to 200. While the corresponding decreasing rate for *T*^2 ^is 14/18-11/18 = 16.7%. It is easy to see that our *X*^2* ^is less liberal than *T*^2 ^as sample size increases.

For Table 2, the total number of significant difference from the nominal level of 0.05 for *X*^2* ^and *T*^2 ^is 17 and 33, respectively. The pair (17, 33) can again be decomposed to (14, 14), (8, 13) and (6, 11) according to n = 50, 100 and 200. The decreasing rate of the number of empirical type I error rates which is significant different from the nominal level of 0.05 for *X*^2* ^is 10/18-1/18 = 50.0% as n increases from 50 to 200. While the decreasing rate for *T*^2 ^is 12/18-10/18 = 11.1%. The decreasing rate of our *X*^2* ^is again more significant than that of *T*^2^.

In Table 3 to 4, the strata increases from 3 to 5. However, the decreasing rates of the number of empirical type I error rates which is significant different from the nominal level of 0.05 for Tables 3 and 4 is very close to that of Tables 1 and 2, respectively. Therefore, we have reason to believe that this decreasing rate is not greatly affected by the number of strata.

Table 5 summarizes the empirical powers for *X*^2* ^and *T*^2^. Here, {*D*_*k*_} are specified under *H*_1 _and we set *D*_*k *_= *D*_0 _+ *δ*(*k *- 1). For *K *= 3, we consider: (i) *D*_0 _= -0.03, *δ *= 0.03 and (ii) *D*_0 _= -0.05, *δ *= 0.05. For *K *= 5, we consider: (i) *D*_0 _= -0.06, *δ *= 0.03 and (ii) *D*_0 _= -0.1, *δ *= 0.05. From the simulation results, we observe both *X*^2* ^and *T*^2 ^perform similarly under the designed parameter settings. In general, powers increase with *n *and *δ*.

**Table 5:** Empirical powers for *X*^2* ^and *T*^2^

n	D	**p**_1+_	**p**_+1_	*X*^2*^	*T*^2^
50, 50, 50	-0.03, 0.0, 0.03	0.5, 0.5, 0.5	0.5, 0.5, 0.5	0.205	0.210
100, 100, 100				0.311	0.306
200, 200, 200				0.560	0.558
50, 50, 50	-0.03, 0.0, 0.03	0.5, 0.4, 0.3	0.5, 0.4, 0.3	0.196	0.203
100, 100, 100				0.360	0.368
200, 200, 200				0.630	0.641
50, 50, 50	-0.03, 0.0, 0.03	0.5, 0.5, 0.5	0.5, 0.4, 0.3	0.197	0.187
100, 100, 100				0.354	0.340
200, 200, 200				0.612	0.612
50, 50, 50	-0.05, 0.0, 0.05	0.5, 0.5, 0.5	0.5, 0.5, 0.5	0.430	0.421
100, 100, 100				0.724	0.720
200, 200, 200				0.958	0.958
50, 50, 50	-0.05, 0.0, 0.05	0.5, 0.4, 0.3	0.5, 0.4, 0.3	0.474	0.483
100, 100, 100				0.797	0.806
200, 200, 200				0.980	0.981
50, 50, 50	-0.05, 0.0, 0.05	0.5, 0.5, 0.5	0.5, 0.4, 0.3	0.457	0.446
100, 100, 100				0.763	0.760
200, 200, 200				0.973	0.973
50, 50, 50, 50, 50	-0.06, -0.03, 0.0, 0.03, 0.06	0.5, 0.5, 0.5, 0.5, 0.5	0.5, 0.5, 0.5, 0.5, 0.5	0.523	0.512
100, 100, 100, 100, 100				0.846	0.841
200, 200, 200, 200, 200				0.993	0.993
50, 50, 50, 50, 50	-0.06, -0.03, 0.0, 0.03, 0.06	0.5, 0.4, 0.3, 0.4, 0.5	0.5, 0.4, 0.3, 0.4, 0.5	0.526	0.526
100, 100, 100, 100, 100				0.854	0.853
200, 200, 200, 200, 200				0.995	0.995
50, 50, 50, 50, 50	-0.06, -0.03, 0.0, 0.03, 0.06	0.5, 0.5, 0.5, 0.5, 0.5	0.5, 0.4, 0.3, 0.4, 0.5	0.535	0.522
100, 100, 100, 100, 100				0.855	0.850
200, 200, 200, 200, 200				0.994	0.994
50, 50, 50, 50, 50	-0.1, -0.05, 0.0, 0.05, 0.1	0.5, 0.5, 0.5, 0.5, 0.5	0.5, 0.5, 0.5, 0.5, 0.5	0.957	0.953
100, 100, 100, 100, 100				1.000	1.000
200, 200, 200, 200, 200				1.000	1.000
50, 50, 50, 50, 50	-0.1, -0.05, 0.0, 0.05, 0.1	0.5, 0.4, 0.3, 0.4, 0.5	0.5, 0.4, 0.3, 0.4, 0.5	0.960	0.960
100, 100, 100, 100, 100				1.000	1.000
200, 200, 200, 200, 200				1.000	1.000
50, 50, 50, 50, 50	-0.1, -0.05, 0.0, 0.05, 0.1	0.5, 0.5, 0.5, 0.5, 0.5	0.5, 0.4, 0.3, 0.4, 0.5	0.957	0.954
100, 100, 100, 100, 100				1.000	1.000
200, 200, 200, 200, 200				1.000	1.000

In view of the above results, we prefer the proposed homogeneity test *X*^2* ^to the traditional *T*^2 ^which is based on the Fisher's test of homogeneity among correlation coefficient.

### Real and hypothetical examples

It is reported that mutations at the cystic fibrosis transmembrane conductance regulator gene (CFTR) cause cystic fibrosis, the most prevalent severe genetic disorder in individuals of European descent. Mateu [15] conducted a worldwide genetic analysis of the CFTR region and analyzed normal allele and haplotype variation at two single-nucleotide polymorphisms (SNPs), namely the T854/*Ava*II (2694 T/G) and TUB20/*PVU*II (4006-200 G/A). The T854 and TUB20 markers can be used to define the core haplotypes since they are diallelic, have presumably much lower mutation rates than the other polymorphisms and the ancestral state can be inferred for them.

Mateu [15] reported the T854-TUB20 haplotype frequencies by 18 populations. After communicating with one of their coauthors (Prof. Kenneth, pers. comm. 1996), it was found that their reported gametic frequencies were actually the maximum likelihood estimates of the gametic probabilities obtained from HAPLO, a software which can be applicable to missing data. In other words, all individuals with results for at least one of the two markers were included to estimate the gametic frequencies and no actual gametic counts were available. To create the gametic counts for each population, we first estimate the total number of participants in each population by the number of individuals who yielded results for at least one of the two markers. The reported gametic frequencies of each population given in Mateu [15] are multiplied to the estimated number of participants of this population and the closest integers are then taken to be the estimated gametic counts. The estimated gametic counts across the 18 populations are reported in Table 6, which is adopted as the real data in all subsequent analysis.

**Table 6:** T854-TUB20 haplotype counts by 18 populations and some related statistics

	Gametic counts				Allele frequencies		Disequilibrium	Estimation	
			
Population	1 - 1	1 - 2	2 - 1	2 - 2	1	1	r	*D'*	*D*
Africa:									
Biaka	5	16	12	29	0.339	0.274	-0.058	-0.132	-0.012
Mbuti	0	14	5	14	0.424	0.152	-0.363	-1.000	-0.064
Tanzanian	0	13	3	20	0.361	0.083	-0.227	-1.000	-0.030
North Africa:									
Saharawi	5	22	12	16	0.491	0.309	-0.263	-0.401	-0.061
Middle East:									
Yemenites	2	29	4	5	0.775	0.150	-0.444	-0.570	-0.066
Druze	2	47	10	4	0.778	0.191	-0.713	-0.786	-0.116
Europe:									
Adygei	1	34	9	5	0.714	0.204	-0.689	-0.860	-0.125
Russians	0	17	10	5	0.531	0.313	-0.718	-1.000	-0.166
Finns	0	23	6	4	0.697	0.182	-0.715	-1.000	-0.127
Catalans	3	53	18	9	0.675	0.253	-0.661	-0.788	-0.135
Basques	4	72	15	17	0.704	0.176	-0.500	-0.701	-0.087
Asia:									
Kazakhs	1	18	2	12	0.576	0.091	-0.155	-0.421	-0.022
Chinese	0	22	1	20	0.512	0.023	-0.158	-1.000	-0.012
Japanese	0	32	0	12	0.727	0	NaN	NaN	0
Yakut	0	18	1	4	0.783	0.044	-0.405	-1.000	-0.034
Pacific:									
Nasioi	1	20	0	22	0.488	0.023	0.158	1.000	0.012
America:									
Maya	2	15	0	31	0.354	0.042	0.282	1.000	0.027
Surui	0	7	0	35	0.167	0	NaN	NaN	0

It is noticed that the gametic counts for the populations of Japanese (14th) and Surui (18th) are (0, 32, 0, 12)' and (0, 7, 0, 35)', respectively and their estimated gametic disequilibrium *D*_*k*_, *D*_*k*,*min *_and *D*_*k*,*max *_are all equal to zero. Therefore, we will exclude these two populations for subsequent homogeneity testings. We consider the following scenarios.

(i) Homogeneity of gametic disequilibrium among the 16 populations (i.e., excluding Japanese and Surui). The statistic value of our proposed *X*^2* ^is 121.35 with *p*-value being less than 0.0001 while that of *T*^2 ^yields 99.64 with *p*-value being less than 0.0001. In this case, both tests reject the homogeneity hypothesis at the 0.05 nominal level.

(ii) Homogeneity of gametic disequilibrium among those populations with the same numbers of participants for both markers T854 and TUB20 (i.e., Mbuti, Yemenites, Druze, Adygei, Catalans, Basques, Chinese, and Nasioi).

Our proposed statistic *X*^2* ^yields 50.56 with *p*-value being less than 0.0001 while *T*^2 ^gives 39.72 with *p*-value being less than 0.0001. Again, both tests suggest rejection of the homogeneity hypothesis at the 0.05 nominal level. Suppose that another research team wants to reconduct the same genetic analysis. In this regard, it is sensible to ask, "How large is the sample size for each population in order to achieve, say, 90% power at the 0.05 nominal level". Based on the present study, we have $\bar{D}$ = (-0.064, -0.066, -0.116, -0.125, -0.135, -0.087, -0.012, 0.012)*'*, ${\bar{p}}_{1+}$ = (0.576, 0.225, 0.222, 0.286, 0.325, 0.296, 0.488, 0.512)*' *and ${\bar{p}}_{+1}$ = (0.849 0.850, 0.810, 0.796, 0.747, 0.824, 0.977, 0.977)*'*. By solving equation (3), n = 157 subjects are required for each of the eight populations under the balanced design.

(iii) Homogeneity of gametic disequilibrium among those populations in Europe.

Our statistic *X*^2* ^yields 7.48 with *p*-value being 0.11 and *T*^2 ^yields 7.26 with *p*-value being 0.12. Both tests do not reject the homogeneity hypothesis at the 0.05 nominal level. In this case, we have evidence to believe that populations in Europe reach their gametic equilibrium.

To end this section, we analyze the hypothetical example of gametic disequilibrium between tow loci (A, B) in ten populations described in Zapata and Alvarez [8]. Here, the gametic counts are simply set by multiplying the haplotype frequencies given in Zapata and Alvarez [8] by 1000. The data are reproduced in Table 7. Obviously, the *r *values are homogeneous across the ten populations. For *D' *values, Zapata and Alvarez [8] utilized the bias-corrected nonparametic bootstrap method to obtain the 95% confidence interval for each *D' *values. Observing that the resultant confidence intervals have no intersection, they concluded that *D' *are heterogeneous. They suggested tests for homogeneity of gametic disequilibrium should be based on *D'*, whose range is allele probability independent, rather than r. Although, the D values in Table 7 seem to be homogeneous, our homogeneity score test yields *X*^2* ^= 33.44 with *p*-value being less than 0.0001. Therefore, our test procedure also suggests the rejection of the homogeneity of gametic disequilibrium across the ten populations. In this case, our test reaches the same conclusion drawn by Zapata and Alvarez [8].

**Table 7:** Hypothetical example of gametic disequilibrium between two loci (A, B) with twoalleles (*A*_0_, *A*_1 _and *B*_0_, *B*_1_, respectively) across ten populations

	Gamete counts				Allele frequencies		Disequilibrium	Estimation	
			
Population	*A*_0 _*B*_0_	*A*_0 _*B*_1_	*A*_1 _*B*_0_	*A*_1 _*B*_1_	*A*_0_	*B*_0_	r	*D'*	*D*
1	495	405	5	95	0.90	0.50	0.300	0.900	0.045
2	540	360	1	90	0.90	0.55	0.300	0.814	0.045
3	479	371	21	129	0.85	0.50	0.300	0.714	0.054
4	460	340	40	160	0.80	0.50	0.300	0.600	0.060
5	671	229	29	71	0.90	0.70	0.300	0.589	0.041
6	539	261	61	139	0.80	0.60	0.300	0.490	0.059
7	615	185	85	115	0.80	0.70	0.300	0.393	0.055
8	373	227	127	273	0.60	0.50	0.300	0.367	0.073
9	403	197	147	253	0.60	0.55	0.300	0.332	0.073
10	325	175	175	325	0.50	0.50	0.300	0.300	0.075

## Discussion

Verification of the homogeneity assumption of gametic disequilibrium across several populations is crucial in gametic disequilibrium analysis. We note that traditional homogeneity test on gametic disequilibrium is based on the Fisher's test of homogeneity among correlation coefficients. However, our simulations demonstrate that this traditional test may not perform satisfactorily. Specifically, it can be very conservative or liberal, for almost all the cases in which the common true gametic disequilibrium D is bounded away from zero. Most importantly, these kinds of conservativeness and liberty can not effectively alleviated with increased sample sizes.

Our proposed large-sample homogeneity score test on gametic disequilibrium across several independent populations requires the count of haplotypes as input. In practice, only genotype data can be obtained in most situations. To employ our method, one can use some haplotyping software, such as PHASE, HAPLOTYPER, to resolve the genotype data as haplotype data. In this way, it separates haplotype phasing and gametic disequilibrium homogeneity test. Naturally, it is more promising to extend our method which can directly handle the genotype data. In this sense, model assumptions are based on genotype data. However, the haplotype phase uncertainty for the double heterozygotes makes the definition of gametic disequilibrium can not be directly expressed by the genotype data even assuming Hardy-Weinberg equilibrium holds. It may severely affect the further derivation of the corresponding score test. Thus, extending our method to handle genotype data is an avenue we intend to explore future.

## Conclusion

In this article, we propose a large-sample homogeneity test on gametic disequilibrium across several independent populations based on the likelihood score theory generalized to nuisance parameters. Our simulation results show that our test is more reliable than the traditional test based on the Fisher's test of homogeneity among correlation coefficients. Although our test may also demonstrate conservativeness and liberty in some cases, unlike the traditional test these issues can be effectively resolved by increasing sample sizes. For design purpose, sample size formula that controls power is derived.

## Authors' contributions

JG initiated the study of homogeneity score test of gametic disequilibrium across strata. XLY drafted the manuscript and conducted the simulation. WQM simplified the proof in the Appendix section and made discussions extensively with XLY. MLT found a real example to apply the proposed method, proposed many constructive comments and widely polished the manuscript. All authors have read and approved the final version of this paper.

## Appendix

### Consistency and the condition to attain asymptotic efficiency for D*

Let *n*_*k *_= *nb*_*k*_, with *b*_*k *_> 0 and *k *= 1, 2,...,*K*. The asymptotic property of D* is obtained under the assumptions that *K *is fixed and *n *approaches infinity (i.e., sufficiently large). The Mantel-Haenszel-type estimator of D* can be rewritten as

$$D^{*}=\sum_{k=1}^{K}\frac{n_{k}^{2}}{x_{01k}x_{10k}}{\hat{D}}_{k}/\sum_{k=1}^{K}\frac{n_{k}^{2}}{x_{01k}x_{10k}},$$

where ${\hat{D}}_{k}=x_{11k}/n_{k}-x_{1+k}x_{+1k}/n_{k}^{2}$. By the Central Limit Theorem, $\sqrt{n}(y_{k}-g_{k})$ has an asymptotic normal distribution *N*(0, Σ_*k*_/*b*_*k*_), where *y*_*k *_= (*x*_00*k*_, *x*_01*k*_, *x*_10*k*_, *x*_11*k*_)/*n*_*k*_, *g*_*k *_= (*p*_00*k*_, *p*_01*k*_, *p*_10*k*_, *p*_11*k*_)*'*, $\Sigma_{k}=diag(g_{k})-g_{k}{g^{\prime }}_{k}$. Let $c_{k}=\frac{\partial {\hat{D}}_{k}}{\partial y_{k}}{|}_{y_{k}=g_{k}}$. By *δ *method, $\sqrt{n}({\hat{D}}_{k}-D_{k})$ follows an asymptotic normal distribution $N(0,{c^{\prime }}_{k}\Sigma_{k}c_{k}/b_{k})$. It is easy to calculate that ${c^{\prime }}_{k}\Sigma_{k}c_{k}=w_{k}(D_{k},p_{1+k},p_{+1k})$. Since *D*_*k *_≡ *D *under *H*_0 _for *k *= 1, 2,...,*K*, we can conclude that D* is a consistent estimate of *D*. Let *w*_*k *_= *w*_*k*_(*D*, *p*_1+*k*_, *p*_+1*k*_), *v*_*k *_= 1/(*p*_01*k*_*p*_10*k*_). Thus, the asymptotic variance of D* under *H*_0 _is given by

$$AsyVar(D^{*})=\frac{\left(\sum_{k=1}^{K}w_{k}v_{k}^{2}/b_{k}\right)}{n{\left(\sum_{k=1}^{K}v_{k}\right)}^{2}}.$$

Let the information matrix with respect to *D*, **p**_1+ _and **p**_+1 _under *H*_0 _be

$$I=\left(\begin{array}{ccccc} \sum_{k=1}^{K}I_{kDD} & I_{1Dp_{1+1}} & I_{1Dp_{+11}} & \cdots & I_{KDp_{+1K}}\\ I_{1Dp_{1+1}} & I_{1p_{1+1}p_{1+1}} & I_{1p_{1+1}p_{+11}} & \cdots & I_{Kp_{1+1}p_{+1K}}\\ I_{1Dp_{+11}} & I_{1p_{1+1}p_{+11}} & I_{1p_{+11}p_{+11}} & \cdots & I_{Kp_{+11}p_{+1K}}\\ \vdots & \ddots & \ddots & & \vdots \\ I_{KDp_{+1K}} & \cdots & \cdots & \cdots & I_{Kp_{+1K}p_{+1K}} \end{array}\right).$$

By inverting the information matrix *I*, we can obtain the asymptotic variance of $\bar{D}$, that is,

$$AsyVar(\hat{D})=\frac{1}{n}{\left(\sum_{k=1}^{K}b_{k}/w_{k}\right)}^{-1}.$$

By Cauchy-Schwarz inequality ${\left(\sum_{k=1}^{K}v_{k}\right)}^{2}\leq (\sum_{k=1}^{K}b_{k}/w_{k})(\sum_{k=1}^{K}w_{k}v_{k}^{2}/b_{k})$, we have *AsyVar*($\bar{D}$) = *AsyVar*(D*). To this end, we obtain the sufficient and necessary condition for the asymptotic efficiency of D*, that is, *w*_*k*_*v*_*k *_= *c*, *k *= 1, 2,...,*K*, where c is a constant independent of all parameters. When *D *= 0, the condition is satisfied. From this, we know that D* is inefficient for general cases.

### A simple expression for $I_{kD|p_{1+k}p_{+1k}}$

For the *k*-th stratum, denote the information matrix with respect to *D*_*k*_, *p*_1+*k *_and *p*_+1*k *_by

$$I_{k}=\left(\begin{array}{ccc} I_{kD_{k}D_{k}} & I_{kD_{k}p_{1+k}} & I_{kD_{k}p_{+1k}}\\ I_{kD_{k}p_{1+k}} & I_{kp_{1+k}p_{1+k}} & I_{kp_{1+k}p_{+1k}}\\ I_{kD_{k}p_{+1k}} & I_{kp_{1+k}p_{+1k}} & I_{kp_{+1k}p_{+1k}} \end{array}\right).$$

According to the property of inverse matrix, $I_{kD|p_{1+k}p_{+1k}}$(*D*_*k*_, *p*_1+*k*_, *p*_+1*k*_) is equal to the reciprocal of the (1, 1) element of $I_{k}^{-1}$. By the property of MLEs, we have

$$\sqrt{n_{k}}({\hat{D}}_{k}-D_{k},{\hat{p}}_{1+k}-p_{1+k},{\hat{p}}_{+1k}-p_{+1k}{)}^{\prime }\overset{d}{\underset{}{\to }}N(0,n_{k}I_{k}^{-1}(D_{k},p_{1+k},p_{+1k})),$$

where ${\hat{D}}_{k},{\hat{p}}_{1+k}=x_{1+k}/n_{k}$ and ${\hat{p}}_{+1k}=x_{+1k}/n_{k}$ are the MLEs of *D*_*k*_, *p*_1+*k *_and *p*_+1*k*_, respectively. Hence, the asymptotic variance of $\sqrt{n_{k}}{\hat{D}}_{k}$ is $n_{k}/I_{kD|p_{1+k}p_{+1k}}(D_{k},p_{1+k},p_{+1k})$. On the contrary, by the Central Limit Theorem, $\sqrt{n_{k}}(y_{k}-g_{k})$ follows an asymptotic normal distribution *N*(0, Σ_*k*_). By *δ *method, we immediately get that $\sqrt{n_{k}}({\hat{D}}_{k}-D_{k})$ follows an asymptotic normal distribution *N*(0, *w*_*k*_(*D*_*k*_, *p*_1+*k*_, *p*_+1*k*_)). Therefore, we can obtain the exact expression $I_{kD|p_{1+k}p_{+1k}}$(*D*_*k*_, *p*_1+*k*_, *p*_+1*k*_) = *n*_*k*_/*w*_*k*_(*D*_*k*_, *p*_1+*k*_, *p*_+1*k*_). Naturally, the expression of $I_{kD|p_{1+k}p_{+1k}}$(*D*, *p*_1+*k*_, *p*_+1*k*_) is just $I_{kD|p_{1+k}p_{+1k}}$(*D*_*k*_, *p*_1+*k*_, *p*_+1*k*_) by substituting *D *for *D*_*k*_.
